# Inclusive research with individuals with Down syndrome at risk for dementia

**DOI:** 10.1590/1980-5764-DN-2024-0280

**Published:** 2025-11-14

**Authors:** Luciana Mascarenhas Fonseca, Ubiratan Tafuri Queiroz, Miron Tafuri Queiroz, Orestes Vicente Forlenza

**Affiliations:** 1 The Krieger Klein Alzheimer’s Research Center, Rutgers Health, New Brunswick, NJ, United States.; 2 Washington State University, Elson S. Floyd College of Medicine, Department Community and Behavioral Health, Spokane WA, United States.; 3 Universidade de São Paulo, Faculdade de Medicina, Hospital das Clínicas, Departamento e Instituto de Psiquiatria, Programa Terceira Idade, São Paulo SP, Brazil.; 4 Universidade de São Paulo, Faculdade de Medicina, Hospital das Clínicas, Departamento e Instituto de Psiquiatria, Laboratório de Neurosciência, São Paulo SP, Brazil.

**Keywords:** Down Syndrome, Intellectual Disability, Alzheimer Disease, Dementia, Healthy Equity, Community-Based Participatory Research, Síndrome de Down, Deficiência Intelectual, Doença de Alzheimer, Demência, Equidade em Saúde, Pesquisa Participativa Baseada na Comunidade

## Abstract

Inclusive research is a relatively new concept that has received attention in recent years as a scientific priority to respond to health disparities and maximize the practical implications of research. This approach involves a partnership between academics and the individuals who are experiencing the problem to be investigated. Despite the high incidence of dementia and asymptomatic Alzheimer’s disease in aging individuals with Down syndrome, inclusive research with this population or those with intellectual disability (ID) at risk of dementia is an area that is little approached and not well documented. Here, we describe the evolution of inclusive research on ID and dementia, some of the challenges and benefits of its implementation, and key aspects to consider when planning such studies. There is an urgent need for national and international guidelines to support inclusive research involving this population. Such frameworks should ensure accessibility, ethical rigor, the meaningful participation of co-researchers, ultimately advancing equity and scientific quality in this underrepresented field.

## RESUMO

Pesquisa inclusiva é um conceito relativamente novo que tem recebido atenção como uma prioridade científica para responder às disparidades de saúde e maximizar as implicações práticas das pesquisas. Essa abordagem envolve uma parceria entre pesquisadores acadêmicos e indivíduos que estão vivenciando o problema a ser investigado. Apesar da alta incidência de demência e doença de Alzheimer assintomática em indivíduos idosos com síndrome de Down, a pesquisa inclusiva com essa população ou com aqueles com deficiência intelectual em risco de demência é uma área pouco abordada e documentada. Neste artigo, descrevemos a evolução da pesquisa inclusiva na área da deficiência intelectual e demência, apresentamos alguns dos desafios e benefícios de sua implementação, assim como os principais aspectos a serem considerados no planejamento desses estudos. Há necessidade de diretrizes nacionais e internacionais para apoiar a pesquisa inclusiva com esta população. Essas normas devem garantir acessibilidade, rigor ético e a participação significativa de copesquisadores, promovendo, assim, equidade e qualidade científica nessa área ainda sub-representada.

## INTRODUCTION

Down syndrome (DS), the most prevalent genetic disorder^1^, is associated with both lifelong intellectual disability (ID)^2^ and increased incidence of cognitive decline due to the development of neuropathological hallmarks of Alzheimer’s disease^3^. Historically, eligibility criteria for clinical trials often directly or indirectly excluded individuals with ID as research participants due to assumptions about their cognitive competence, ethical concerns, and systemic biases^4^. Individuals with disability, including those with DS, are now considered a health disparity population^5^, and recent efforts encourage the adoption of community engagement requirements to maximize the inclusion of such participants in research^5^. While both participatory and inclusive research involve collaboration, inclusive research goes a step beyond the involvement of individuals with disability as research participants by establishing a partnership between them and the academics performing the study. As such, inclusive research specifically centers people with ID as co-researchers, contributing meaningfully to the design, implementation, and dissemination of research. This approach emphasizes their leadership and expertise by experience and marks an evolution toward greater epistemic justice within disability research. Recently, employing inclusive approaches has been considered a priority in studies of human subjects as a way to address health disparities and bridge important gaps between research, clinical work, and community implementation of scientific outcomes^6^. Moreover, studies including individuals with ID and individuals with dementia as co-investigators have proven to be feasible, valuable, and beneficial for the development of the research, as well as important for broadening the professional and personal experiences of the academic researchers involved^7,8,9^. The aim of this paper is to critically examine the principles and practices of inclusive research involving people with ID and the field’s potential to amplify marginalized voices. By tracing its evolution, interrogating key concepts, and analyzing implementation challenges, the paper addresses existing gaps to inform more equitable research approaches.

## EVOLUTION OF INCLUSIVE ID RESEARCH

As a result of ongoing developments in the health field and the emerging empowerment of individuals with ID to make their own health decisions, they are now increasingly seen as partners in the health system^10^. In addition, given the emphasis on individual health rights, these individuals are currently and gradually being included as collaborators in health research^11^. The United Nations Convention on the Rights of Persons with Disabilities (UN-CRPD) ensured that people with disabilities have the right to make decisions about their own lives and have the necessary support to do so^12^. The convention culminated in important legal developments in several countries. In Brazil, the convention was ratified in 2007 and, based on the convention, the Brazilian Inclusion Law was created in 2015, reinforcing and legitimizing the rights of individuals with disabilities and aiming to ensure their ability to exercise their rights under equal conditions^13^. The concept of “nothing about us, without us” has become a central motto of this self-advocacy movement. With the emphasis on individuals with disabilities having the right to make their own health decisions, active participation of this population in health has been extended through inclusive research. However, a consensus statement on inclusive research recently published by a partnership between individuals with ID and academics highlighted the fact that people with ID are not yet structurally involved in health research^11^. Building on these developments, inclusive research is fundamentally rooted in the principles of autonomy, self-determination, and shared decision-making. Facilitating these principles requires creating accessible communication methods, providing appropriate supports, and fostering collaborative environments that empower individuals with ID to actively contribute to all stages of research. These approaches help to dismantle traditional power imbalances and ensure that research reflects their lived experiences and priorities. For example, in Arcoverde, Brazil, a participatory video project engaged individuals with disabilities as co-researchers and illustrated how inclusive research can embody right-based ideals, but also expose structural limitations such as inadequate institutional support, the critical role of academic researchers being to ensure co-participants with disability are not left with specific actions and limited accessibility within healthcare and academic systems^14^.

Inclusive research is grounded in the social model of disability, which emphasizes that disability arises not solely from individual impairments but from societal barriers—attitudinal, structural, and communicational—that must be identified and addressed throughout the research process. Inclusive research on ID generally implies a partnership between the individuals with ID and the academics conducting the study, with both parties contributing to the development of studies and the dissemination of findings. It also includes consideration of the interests of individuals with ID in their own voices^15^. There is a pluralistic approach to the inclusion of individuals with ID in research, with three main study designs that have been conceptualized in a comprehensive review of inclusive research in ID: the first includes individuals with ID providing advice on research methods and procedures, the second includes these individuals in leading and controlling the study; and the third includes individuals with and without disability working together, considering interests and different skills^16^.

Inclusive research has been widely recognized for the past three decades as a novel approach to conducting research^10,11^. In 2012, the British Journal of Learning Disabilities published a special issue on inclusive research in ID in which many of the articles were co-authored by individuals with ID, and the peer-review team also included researchers with ID working alongside academics^17^. In 2014, the Journal of Applied Research in Intellectual Disabilities released a special issue updating the topic of inclusive research and fostering discussions of the challenges of its implementation^15^. In 2022, the Journal of Social Sciences published a special issue on inclusive research with a focus on ID^18^. While there have clearly been advancements in the field, inclusive research specifically with individuals with Down syndrome or those with ID and risk for dementia has yet to be addressed and documented. The last Summit on ID and Dementia highlighted the importance of involving individuals with ID and dementia as participants in research^19^, suggesting the need for strategies that promote citizenship, social inclusion, and the rights of people with ID and dementia^20^. Inclusive research that effectively involves individuals with DS and dementia would certainly be a great first step towards substantiating the real meaning of citizenship and social inclusion for this population.

## UNDERLYING CONCEPTS OF INCLUSIVE RESEARCH

From a practical point of view, inclusive research presupposes other more basic concepts of inclusion, such as consideration of the health needs of individuals with ID, their recruitment as research participants^21^, and appropriate contemplation of their autonomy and capacity to assent to participate in research. Many current studies with individuals with ID do not include self-reported data, although these individuals are able to provide relevant information about their own health, which sometimes differs from the opinion of their informants^22,23^. Research has also demonstrated that participants with mild to moderate dementia can provide assent and reliably self-report information^24,25^. However, due to the presence of premorbid intellectual deficits and given the complex institutional review board requirement of informed consent forms containing all the information that needs to be considered by the patient, individuals with ID are not always able to legally sign their consents. Regardless of the local laws on capacity to consent that should be followed, assent to participate should also be sought from all participants with ID and systematically described, no matter the level of disability or the presence of cognitive decline and/or dementia. There is a need to accommodate and address any specific difficulties in being able to consent or assent to participate in research. This includes procedures being appropriately adapted in culturally sensitive ways to meet individual needs and overcome any difficulties in understanding the content. For example, sustained and dynamic consent should be examined across different phases of cognitive decline to ensure ongoing ethical participation. Such adaptations could consist of, for example, the use of adapted technology, concrete and simple language, and illustrations. Inclusive research challenges traditional academic hierarchies by actively redistributing power, fostering ethical reflexivity among researchers, and amplifying the voices of individuals with ID as equal contributors, while avoiding tokenism, when involvement is superficial rather than meaningful.

## CHALLENGES OF EFFECTIVE IMPLEMENTATION

Some of the main challenges of implementing inclusive research are adequately identifying barriers and facilitators, adapting to different levels of cognitive ability and specific cognitive domain deficits, granting appropriate funding for training, and adapting environment assistive technology, if needed. Indeed, for a group of academic researchers working on inclusive research in dementia, the main challenges described were the additional time required for planning and adapting the research protocol to include co-researchers with dementia, as well as increased costs due to the extra training required and the need for adaptive support^7,8^. Therefore, research studies that include individuals with ID need to plan for the early identification of barriers and facilitators, develop ways to appropriately address barriers, identify the real health equity demand by considering the voices of individuals with ID, create research procedures and materials that engage these individuals in a respectful way, and collect data that is representative, reliable, and valid^26^. Some practical recommendations to support these points include collaboratively developing stepwise guidance with clear instructions to effectively conduct inclusive research, ensuring ongoing monitoring of each stage, and creating templates for inclusive interview protocol. Inclusive research also implies that researchers who do not have ID will provide sensitive support to those with ID, which will involve balancing the right amount of support needed with maintaining the autonomy and self-interest of the individual with ID. While many challenges of inclusive research apply broadly, working with people with ID and dementia requires additional considerations, such as need-tailored communication strategies, and heightened ethical vigilance to ensure respect and autonomy throughout the research.

**Figure 1.**
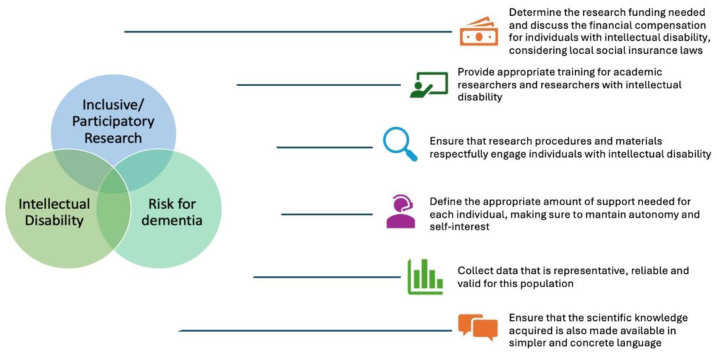
Schematic overview of key aspects to consider when planning inclusive research with individuals with intellectual disability at risk of dementia.

When conducting inclusive research, it is also important that the research is clear in the scope of its development and that the impact of its added value is disseminated via publications in both scientific and non-scientific media. Scientific knowledge is often published in technical language that is not always accessible to lay or non-academic individuals. With appropriate support, participants with DS at risk for dementia can participate in the development of manuscripts, as there are examples of scientific publications with co-authors with ID^27,28,29^. Developing translational results using lay language is also important to ensure that study information is available to co-researchers and the general population. Moreover, inclusive research must recognize that factors such as gender, race, and socioeconomic status intersect with ID and influence individuals’ experiences, creating the need for tailored approaches to consent, communication, and participation. A schematic overview of key aspects to consider when planning inclusive research with individuals with ID at risk of dementia is presented in Figure 1.

To address challenges in inclusive research, this study involved collaboration with co-researchers with and without DS using adapted communication, shared decision-making, and co-authorship, alongside producing a simplified lay version of the manuscript to ensure accessibility and meaningful participation. Many of the disadvantages of inclusive research—such as unclear role definitions, communication barriers, inconsistent levels of involvement, and difficulties in ensuring ethical and meaningful participation—result from the lack of clear guidelines for inclusive research methodology and the dearth of training on both sides. Thus, greater clarity and organization of the objectives of the partnership established between the academic researchers and researchers with ID are necessary for an effective experience. Both academic and non-academic researchers performing inclusive research with subjects with dementia or ID report that the experience of inclusive research expands their theoretical knowledge and also increases their professional competence in working with this population^7,28,29^. A key methodological distinction between inclusive research and participatory action research (PAR) is that inclusive research involves leadership and active participation of people with ID throughout the research, whereas PAR often involves a broader community participation^16^. When implemented effectively, inclusive research enriches the academic community by fostering diversity, enhancing the relevance and applicability of research questions, and improving the quality and impact of scientific knowledge through the inclusion of lived experience.

## THE NEED FOR INCLUSIVE RESEARCH

The inclusion of people with DS and ID in the development of research methodology and project implementation—along with the incorporation of stakeholders’ feedback and ensuring accessible language in dissemination—contributes to the quality and reliability of the research. This approach supports the well-being of those involved, improves reliability of the data collected, and increases the real-world applicability of clinical outcomes. Furthermore, inclusive research represents an opportunity to better qualify research questions by incorporating the perspectives and lived experiences of people with DS and their families into all stages of the investigative process. The involvement of individuals with ID and their relatives brings distinct perspectives and needs to the research process, which may not always align; inclusive research must ensure that the voices of people with ID are prioritized and not overshadowed by proxy representation. This active participation highlights priority issues, ensuring that the development of novel knowledge and the advancement of science meet the real needs of these people. Furthermore, the integration of accessible and collaborative methodologies will enrich our understanding of the biopsychosocial factors associated with dementia in DS, improving the translation of scientific discoveries into ecological solutions that are applicable in real contexts. Accessible and collaborative methodologies aim to remove barriers to participation by adapting research processes to the communication and cognitive needs of individuals with ID. Examples include the use of easy read materials, visual aids, participatory video, photo-voice, and co-designed interview guides. Projects such as the Promoting Effective and Empowered Research (PEER) project^30^ have demonstrated how co-researcher training, shared decision-making, and ongoing support can enable meaningful participation.

In conclusion, inclusive research with people with DS that are at risk for dementia combines social, political, and cultural changes with visions of health, equity, and the right to self-determination. Effective and sensitive implementation of research that not only considers the opinions of these people but also includes population representatives throughout the research process, including defining the research problem, developing and implementing protocols, and disseminating results, can result in immeasurable scientific advancements and effectively meaningful benefits that address their needs. National and international guidelines are urgently required. Future empirical studies evaluating their outcomes will help guide these efforts. These should address methodological standards, accessibility, consent procedures, and the meaningful involvement of co-investigators with ds and dementia. Establishing such frameworks will promote consistency, accountability and greater equity in research.

## Data Availability

No new data were generated or analyzed in this study.
